# Supplementary material for: Molecular basis for the disruption of Keap1–Nrf2 interaction via Hinge & Latch mechanism
Source: Commun Biol. 2021 May 14;4:576. doi: 10.1038/s42003-021-02100-6 (PMC8121781; doi:10.1038/s42003-021-02100-6)
Supplement: Supplementary file 2 — Supplementary Information [file 42003_2021_2100_MOESM2_ESM.pdf]

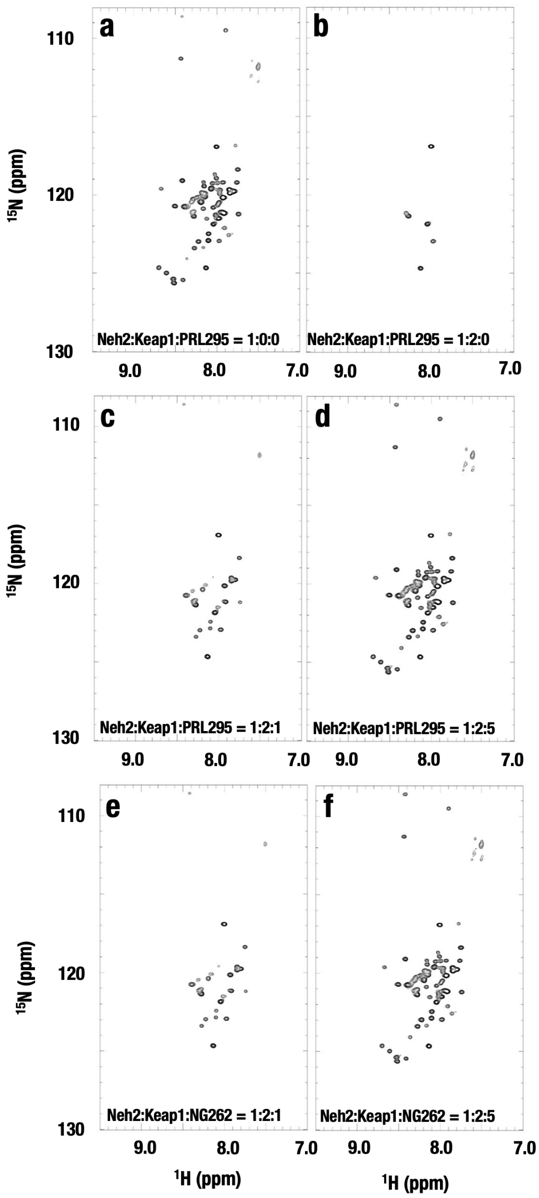

Supplementary Figure 1. Titration of Neh2-Keap1 complex with pharmacological Keap1-Nrf2 PPI inhibitors. 2D TROSY-HSQC spectrum of $^{13}C/^{15}N$-labeled Neh2 in the absence (**a**) or presence (**b**) of 2-fold excess of Keap1. 2D TROSY-HSQC spectrum of $^{13}C/^{15}N$-labeled Neh2 in the presence of 2-fold excess of Keap1 after the addition of equal amount (**c**) or 5-fold excess amount (**d**) of PRL295. 2D TROSY-HSQC spectrum of $^{13}C/^{15}N$-labeled Neh2 in the presence of 2-fold excess of Keap1 after the addition of equal amount (**e**) or 5-fold excess amount (**f**) of NG262.

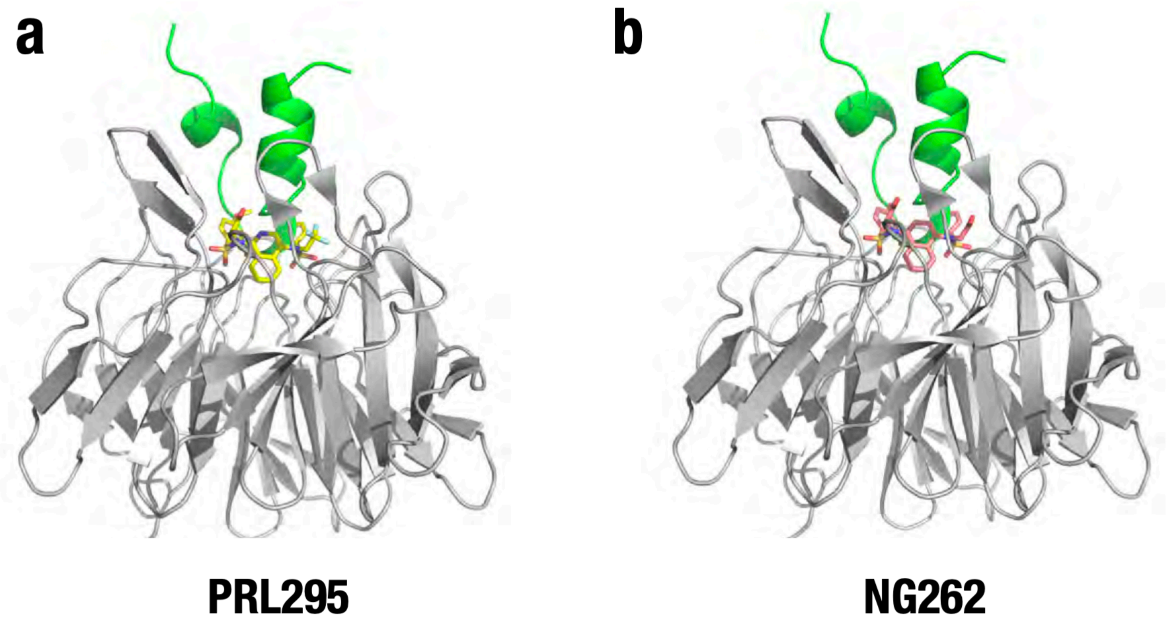

Supplementary Figure 2. Merged images of DLGex with PRL295 (a) or NG262 (b) associating with Keap1 pocket.

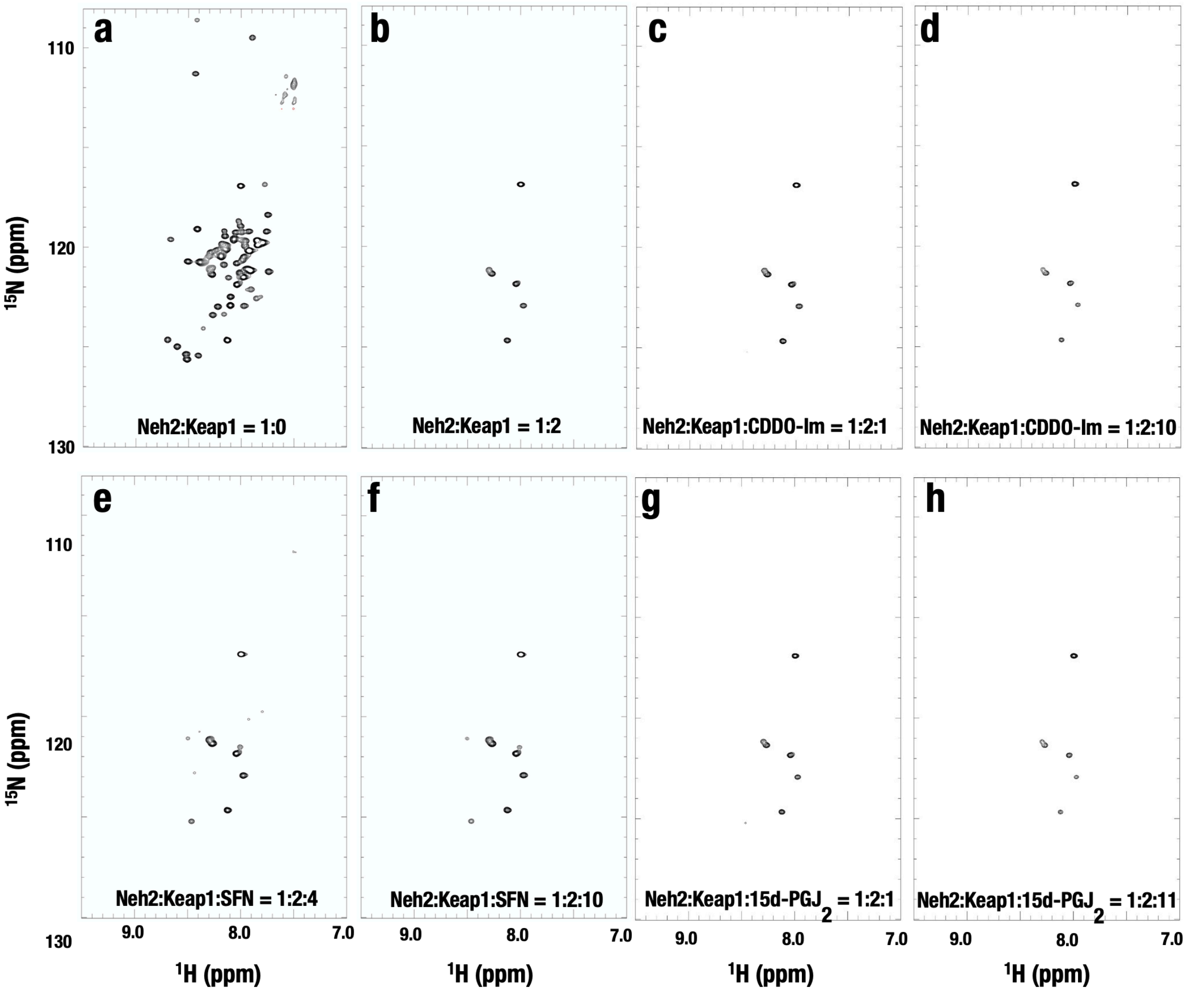

Supplementary Figure 3. Titration of Neh2-Keap1 complex with electrophilic Nrf2 inducers. 2D TROSY-HSQC spectrum of $^{13}C/^{15}N$-labeled Neh2 in the absence (**a**) or presence (**b**) of 2-fold excess of Keap1. 2D TROSY-HSQC spectrum of $^{13}C/^{15}N$-labeled Neh2 in the presence of 2-fold excess of Keap1 after the addition of equal (**c**) or 10-fold excess amount (**d**) of CDDO-Im. 2D TROSY-HSQC spectrum of $^{13}C/^{15}N$-labeled Neh2 in the presence of 2-fold excess of Keap1 after addition of 4-fold excess (**e**) or 10-fold excess (**f**) of SFN. 2D TROSY-HSQC spectrum of $^{13}C/^{15}N$-labeled Neh2 in the presence of 2-fold excess of Keap1 after the addition of equal amount (**g**) or 11-fold excess amount (**h**) of 15d-PGJ_2.

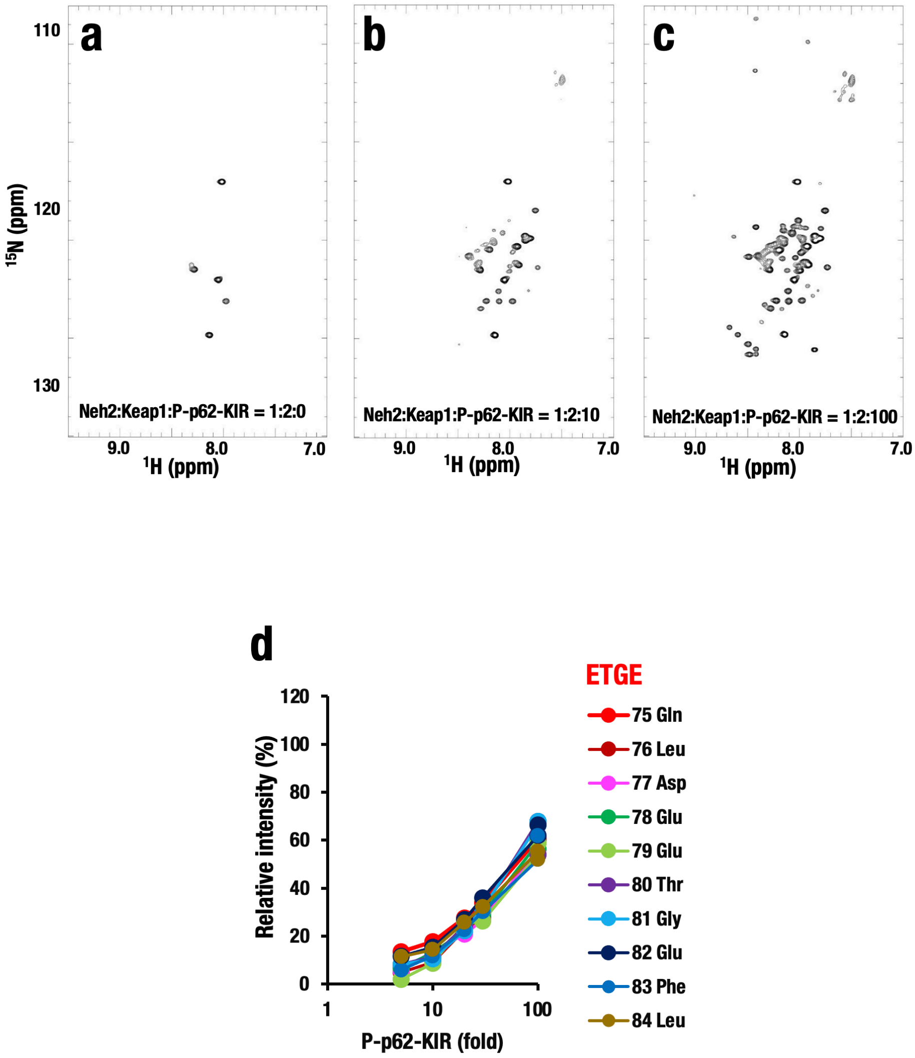

Supplementary Figure 4. Titration of Neh2-Keap1 complex with P-p62-KIR peptide. (**a-c**) 2D TROSY-HSQC spectrum of $^{13}C/^{15}N$-labeled Neh2 in the presence of 2-fold excess of Keap1 in the absence of the peptide (**a**), or in the presence of 10-fold (**b**) or 100-fold P-p62-KIR peptide (**c**). (**d**) Relative peak intensity of ETGE motif by the addition of P-p62-KIR peptide.

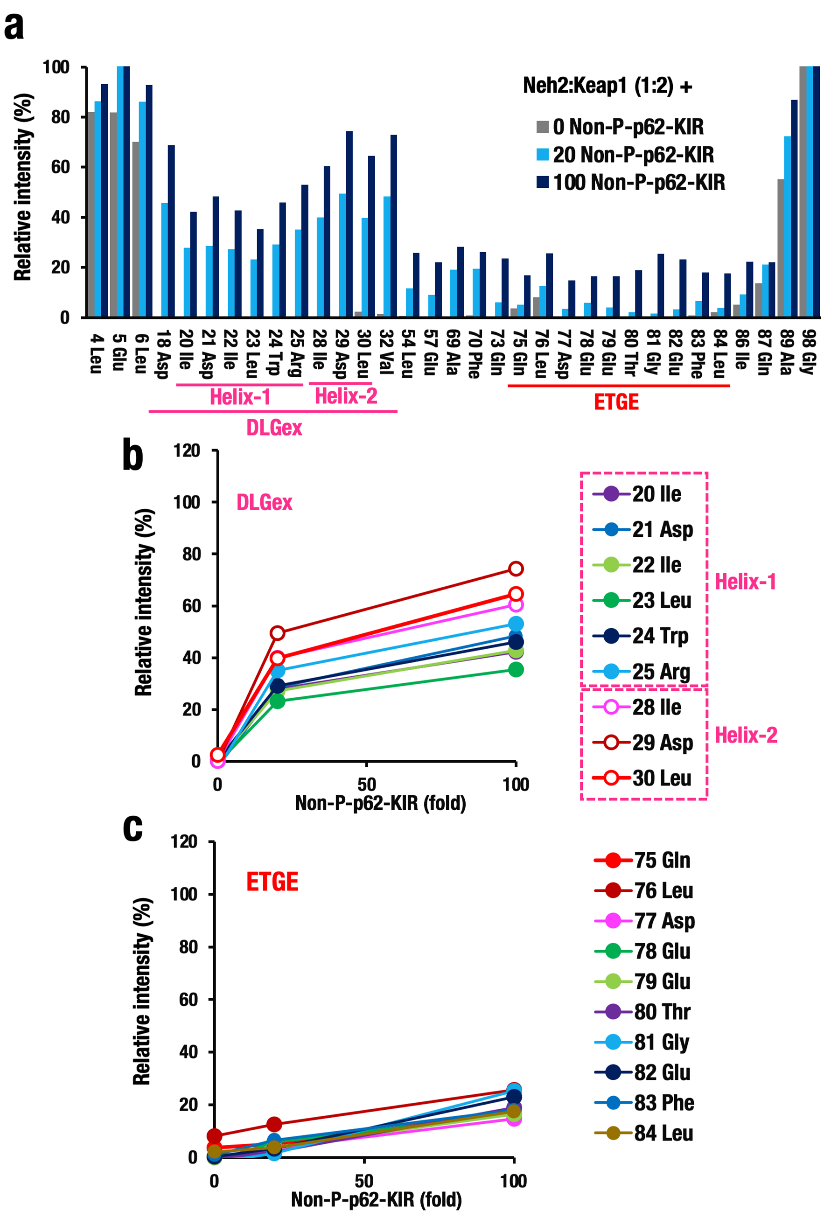

Supplementary Figure 5. Titration of Neh2-Keap1 complex with Non-P-p62-KIR peptide. (**a**) Relative peak intensity of each amino acid residue of Neh2 in the Neh2-Keap1 complex (1:2) in the titration experiments by the addition of 20- and 100-fold excess of Non-P-p62-KIR peptides. (**b**) Relative peak intensity of each amino acid residue within the DLGex motif by the addition of Non-P-p62-KIR peptides. Closed and open circles indicate the amino acids corresponding to Helix-1 and Helix-2, respectively. (**c**) Relative peak intensity of each amino acid residue within the ETGE motif by the addition of Non-P-p62-KIR peptide.
