# Supplementary material for: Molecular basis for the disruption of Keap1–Nrf2 interaction via Hinge & Latch mechanism
Source: Commun Biol. 2021 May 14;4:576. doi: 10.1038/s42003-021-02100-6 (PMC8121781; doi:10.1038/s42003-021-02100-6)
Supplement: Supplementary file 3 — Descriptions of Additional Supplementary Files [file 42003_2021_2100_MOESM3_ESM.pdf]

Descriptions of Additional Supplementary Files

Supplementary data 1
Description: All source data underlying the graphs presented in the main figures.
